# Secular trends in low birth weight and child undernutrition in West Africa: evidence from complex nationwide surveys, 1985–2019

**DOI:** 10.1017/S1368980022000155

**Published:** 2022-09

**Authors:** Duah Dwomoh, Christian Sewor, Seidu Awal Mohammed, Samuel Annim, Saverio Stranges, Ngianga-Bakwin Kandala, A Kofi Amegah

**Affiliations:** 1 Public Health Research Group, Department of Biomedical Sciences, University of Cape Coast, Private Mail Bag, Cape Coast, Ghana; 2 Department of Biostatistics, School of Public Health, University of Ghana, Legon – Accra, Ghana; 3 Department of Clinical Nutrition and Dietetics, University of Cape Coast, Cape Coast, Ghana; 4 Department of Applied Economics, School of Economics, University of Cape Coast, Cape Coast, Ghana; 5 Ghana Statistical Service, Accra, Ghana; 6 Department of Epidemiology and Biostatistics and Africa Institute, Western University, London, ON, Canada; 7 Department of Population Health, Luxembourg Institute of Health, Strassen, Luxembourg; 8 University of Warwick, Division of Health Sciences, Warwick Medical School, Coventry, UK; 9 University of the Witwatersrand, Division of Epidemiology and Biostatistics, School of Public Health, Johannesburg, South Africa

**Keywords:** Children, Stunting, Wasting, Underweight, Anaemia, Low Birth Weight, West Africa

## Abstract

**Objective:**

We present prevalence estimates and secular trends of stunting, wasting, underweight, and anaemia among children under 5 years of age and low birth weight (LBW) over the period 1985–2019 in West Africa (WA).

**Design:**

Analysis of Demographic and Health Survey (DHS) and World Bank data. DerSimonian–Laird random effect model with the Knapp–Hartung adjustment to the standard error was used to derive overall prevalence estimates. We used fixed effect ordinary least square regression models with cluster robust standard error to conduct time trends analyses.

**Setting:**

West Africa.

**Participants:**

Children aged 0 to 59 months.

**Results:**

Three distinct periods (1986–1990, 1993–1996 and 1997–2000) of sharp increases in prevalence of all outcomes was observed. After the year 2000, prevalence of all outcomes except LBW started to decline with some fluctuations. LBW prevalence showed a steady increase after 2000. We observed a decline in prevalence of stunting (*β* = –0·20 %; 95 % CI –0·43 %, 0·03 %), log-wasting (*β* = –0·02 %; 95 % CI –0·02 %, –0·01 %), log-underweight (*β* = –0·02 %; 95 % CI –0·03 %, –0·01 %) anaemia (*β* = –0·44; 95 % CI –0·55 %, –0·34 %), and an increase in LBW (*β* = 0·06 %; 95 % CI –0·10 %, 0·22 %) in WA over the period. Pooled prevalence of stunting, wasting, underweight, anaemia and LBW in WA for the period 1985–2019 was 26·1 %, 16·4 %, 22·7 %, 76·2 % and 11·3 %, respectively.

**Conclusions:**

Child undernutrition prevalence varied greatly between countries and the year cohorts. We observed marginal reductions in prevalence of all outcomes except anaemia where the reductions were quite striking and LBW where an increase was noted. There is the need for more rigorous and sustained targeted interventions in WA.

Some progress has been made over the past few decades in addressing low birth weight (LBW) and reducing child undernutrition in sub-Saharan Africa (SSA), but it has been slow compared to other geographical regions of the world. Between 2000 and 2019, stunting prevalence in Africa declined from 37·8 % to 21·8 %^(1)^. The Asia region for instance reduced stunting prevalence by over 40 % during the same period. Although the prevalence of stunting has decreased, the number of stunted children in Africa has increased from 49·7 million in 2000 to 57·5 million in 2020^(1)^. In fact, Africa is the only region where the number of stunted children has risen. Within the African region, progress has been slower in West Africa (WA) than the other subregions. In WA, 18 million children were stunted, the second highest in the African region, with a prevalence rate of 27·7 % in 2019^(1)^ More than a quarter of wasted children in the world, translating into 12·7 million, reside in Africa with WA recording the highest prevalence of wasting (7·5 %) in Africa^(1)^. About 20 % of children under 5 years of age in WA are underweight and is the highest in SSA^(2)^. The prevalence of LBW in WA is 15·2 % and is the highest in Africa^(3)^. According to 2011 estimates, prevalence of severe anaemia is less than 2·5 % in all geographical regions of the world, except among children in Central Africa and WA (9·7 %) and East Africa (10·2 %)^(4)^. Fifty-three million children in Central Africa and WA have been reported to be anaemic^(4)^. According to a recent study, the prevalence of iron deficiency anaemia among African children may be substantially higher than current estimates by WHO suggests^(5)^.

LBW and undernutrition are associated with a range of both short- and long-term sequelae including higher risk of morbidity in childhood, adult-onset chronic conditions such as high glucose concentrations, blood pressure, hyperlipidemia and CVD, impairment of cognitive development and behavioural function, and death and disability of millions of children every year^(6–12)^.

Despite the huge investments by governments and the several efforts put forth by non-governmental organisations and multilateral agencies in WA to reduce the burden of child undernutrition, iron deficiency anaemia and LBW, the problem still persist with lack of clarity on the actual prevalence and trend in the subregion. This is because majority of the prevalence estimates combined by meta-analytic studies are from individual studies that are not nationally representative and suffer from random and systematic error. The UNICEF/WHO/World Bank Group Joint Child Malnutrition Estimates uses nationally representative country-level dataset but do not examine country-wide variations and also do not provide estimates for iron deficiency anaemia and LBW. We therefore investigated secular trends of child undernutrition (stunting, wasting, underweight and anaemia) and LBW for the period 1980 to 2020 (four decades) in WA leveraging prevalence estimates from Demographic and Health Surveys (DHS) which are nationally representative surveys and are standardised across countries to make available the actual prevalence and trend of these adverse child outcomes whilst also examining country-wide variations. We complemented these data with estimates from the World Bank database which are also nationally representative.

Child undernutrition indicators are used as proxy for broader human development in low and middle income countries (LMIC) and as a result summary estimates are routinely needed to evaluate the state of human development in these countries. The estimates from our study will also serve as a guide for assessing impact of the several nutritional interventions implemented in WA over the years as well as driving a rigorous region-wide intervention programme for tackling undernutrition in the region. Furthermore, for West African countries to successfully meet the Sustainable Development Goal (SDG) 2.2 (malnutrition targets), countries and multilateral agencies require reliable estimates from standardised surveys to aid planning and national policy decision-making.

## Methods

### Data sources

We assembled data from 60 DHS (https://dhsprogram.com/) reports and the World Bank data repository (https://databank.worldbank.org/home.aspx) from 1985 to 2019 in 14 West African countries. Prevalence estimates of LBW, stunting, wasting, underweight and anaemia during the period were extracted from the DHS reports and the World Bank data repository to create a dataset with a maximum of 60 data points from 14 countries. The time period for the conduct of DHS surveys varied across the 14 countries within a time interval of 5 years. However, in some countries, the surveys have not been conducted consistently. We therefore complemented the DHS data with the yearly estimates from the World Bank repository where necessary. For the meta-analysis, only estimates from the DHS reports were used. This is because the DHS reports had information on the weighted and unweighted sample of children interviewed and also, the prevalence estimates were reported with their corresponding standard errors and 95 % confidence interval (CI). The World Bank repository estimates, however, had no such information to complement the point estimates.

### Outcomes of interest

The outcomes of interest were LBW, and childhood stunting, wasting, underweight and anaemia. LBW was classified as birth weight less than 2·5 kg^(13)^. Children were classified as stunted if their height for age z-scores (HAZ) was below –2 standard devaiations (sd) from the median of the reference population^(14)^. Children were classified as wasted if their weight for height z-scores (WHZ) was below –2 sd from the median of the reference population^(14)^. Children were classified as underweight if their weight for age z-scores (WAZ) was below –2 sd from the median of the reference population^(14)^. The nutritional assessment was conducted among children aged 0 to 59 months. Children aged 6 to 59 months with hemoglobin (Hb) levels less than 11·0 g/dl were classified as anaemic^(15)^. DHS does adjust Hb levels for altitude of the dwelling above 1000 m (3300 feet) using data on cluster altitude. The data sources do not measure Hb levels among children less than 6 months of age. This is because this group of children have higher levels of Hb at birth and just after birth and may distort the indication of anaemia prevalence.

The DHS reports have information on nutritional status of children under 5 years of age which the survey computes from measurement of height and weight of the children using a Shorr Productions measuring board and SECA 878 digital scale, respectively, together with information on ages of the children. The World Bank repository collects information on prevalence estimates from different sources including the DHS and UNICEF.

### Secular trend analysis

The data assembled from the DHS reports and World Bank data repository for each outcome were tested for normality and where they were found not to be normally distributed, they were log-transformed to approximate normality before the statistical analyses.

To adjust for correlation in the outcome measures, we used fixed effect ordinary least square regression models with clustered robust standard errors to conduct time trend analyses on the prevalence estimates between 1985 and 2019 to obtain the average annual percentage change in prevalence of the outcomes of interest. We stratified the analysis into three periods; 1990–1999, 2000–2009 and 2010–2019. In the ordinary least square regression models fitted, the prevalence estimates represented the response variable with the year of survey as the predictor variable. We conducted sensitivity analysis using different variant of the outcome model specification.

### Meta-analysis

We estimated an adjusted prevalence for each outcome across WA taking into account between- and within-country variability. The random effects meta-analysis with DerSimonian–Laird method^(16)^ was used to obtained a summary prevalence estimate and its corresponding 95 % CI for the West African region. For each outcome, we combined all the available prevalence estimates during the study period for the countries. The DerSimonian–Laird method takes into account within-country variability attributable to the different time points of the surveys conducted in countries and between-study variability owing to different prevailing conditions in countries. The DerSimonian–Laird method does not make any assumptions about the distribution of random effects.

We applied the Knapp–Hartung adjustment^(17)^ to adjust for the standard error of the summary prevalence estimate. The Knapp–Hartung adjustment improves precision of the estimated between-study variance associated with using small samples. Publication bias owing to prevalence estimate for an outcome of interest not being estimated for a particular country in a particular survey year or the survey not being conducted during that survey year was investigated by inspecting funnel plot for asymmetry and confirming with the Egger’s test. A *P*-value < 0·05 on the Egger test was deemed to be indicative of statistically significant publication bias.

Heterogeneity was assessed using the Cochran’s χ^2^ test and quantified using the *I* ^*2*^ statistic. The null hypothesis of the heterogeneity test was that all surveys conducted at different time points across the various countries share a common prevalence estimate for the outcome of interest. The *I* ^*2*^ statistic estimates the percentage of total variation across different DHS surveys due to true between-study differences rather than chance. *I* ^*2*^ values greater than 60–70 % indicate the presence of substantial heterogeneity^(18)^.

Meta-regression was conducted to identify the source of heterogeneity, that is, we investigated whether between-study heterogeneity can be explained by one or both covariates (year of survey and country of survey). All statistical analyses were conducted using Stata version 16 (StataCorp LLC).

## Results

Table 1 presents the sources of data and the number of data points used in assessing secular trend of the outcomes of interest in the West African countries. Data from the DHS reports and World Bank data repository spanned the period 1985 and 2019. The number and proportion of data points from the DHS reports and World Bank data repository were 60 (14·8 %) and 346 (85·2 %), respectively. The total number of data points obtained for each country ranged between 28 and 30 for the period 1985 to 2019.

**Table 1:** Characteristics of data points assembled for the trend analysis

Country	DHS survey(s) assessed	Number of data points from DHS		Survey years of World Bank data repository	Number of data points from World Bank data repository		Total number of data points
Benin	1996, 2001, 2006, 2011, 2017	5		1990–1995, 1997–2000, 2002–2005,	24		29
				2007–2010, 2012–2013, 2015–2016			
				2017–2018			
Burkina Faso	1993, 1998, 2003, 2010	4		1990–1992, 1994–1997, 1999–2002	25		29
				2004–2009, 2011–2018			
Cote d’Ivoire	1994, 1998, 2011	3		1986–1993, 1995–1997, 1999–2010, 2012–2016	25		28
Gambia	2013	1		1990–2012, 2014–2016, 2018	28		29
Ghana	1988, 1993, 1998, 2003, 2008, 2014	6		1990–1992, 1994–1997, 1999–2002	23		29
				2004–2007, 2009–2013, 2015–2017			
Guinea	1999, 2005, 2012, 2018	4		1990–1998, 2000–2004, 2006–2011	25		29
				2013–2017			
Liberia	1986, 2007, 2013	3		1990–2006, 2008–2012, 2014–2016	25		28
Mali	1987, 1995, 2001, 2006, 2012, 2018	6		1990–1994, 1996–2000, 2002–2005	24		30
				2007–2011, 2013–2017			
Mauritania	2001	1		1990–2000, 2002–2018	28		29
Niger	1992, 1998, 2006, 2012	4		1985–1991, 1993–1997, 1999–2005			
				2007–2011, 2013–2018			
Nigeria	1990, 1999, 2003, 2008, 2013, 2018	6		1991–1998, 2000–2007	22		28
				2009–2012, 2014–2016			
Senegal	1986, 1992, 1997, 1999, 2005, 2010, 2012, 2014, 2015, 2016, 2017, 2018	12		1990–1991, 1993–1996, 2000–2004	18		30
				2006–2009, 2011, 2013, 2019			
Sierra Leone	2008, 2013	2		1989–2007, 2009–2012, 2014–2019	28		30
Togo	1988, 1998, 2013	3		1990–1997, 1999–2012, 2014–2017	26		29
		60	14·8 %		346	85·2 %	406

Table 2 presents time trends analyses on the outcomes of interest between 1985 and 2019 in WA. The results of the trend analysis from the fixed effect ordinary least square regression model with cluster robust standard error showed a decline in prevalence of log-wasting (*β* = –0·02 %; 95 % CI –0·02 %, –0·01 %), log-underweight (*β* = –0·02 %; 95 % CI –0·03 %, –0·01 %) and anaemia (*β* = –0·44 %; 95 % CI –0·55 %, –0·34 %) among children under 5 years of age. Prevalence of stunting also decreased (*β* = –0·20 %, 95 % CI –0·43, 0·03) with prevalence of LBW increasing over the study period (*β* = 0·06 %, 95 % CI –0·10 %, 0·22 %). The 95 % CI of the average annual percentage change for both stunting and LBW, however, included the null value. Sensitivity analysis using different variant of the outcome model specification showed similar trend for the five outcome measures over the period.

**Table 2:** Time trends analyses of low birth weight and childhood undernutrition between 1985 and 2019 in West Africa

Outcomes			Sensitivity analysis					
	Fixed effect model with cluster robust standard error		Random effect model with cluster robust standard error		Mixed effect model with cluster robust standard error		Generalised estimating equation with Gaussian link and exchangeable correlation structure with robust standard error (population average models)	
	*β*	95 % CI	*β*	95 % CI	*β*	95 % CI	*β*	95 % CI
Stunting	–0·20	–0·43, 0·03	–0·19	–0·40, 0·01	–0·19	–0·40, 0·01	–0·20	–0·40, 0·01
Log-wasting	–0·02	–0·02, –0·01	–0·02	–0·02, –0·01	–0·02	–0·02, –0·01	–0·02	–0·02, –0·01
Log-underweight	–0·02	–0·03, –0·01	–0·02	–0·02, –0·01	0·02	–0·02, –0·01	–0·02	–0·02, –0·01
Low birth weight	0·06	–0·10, 0·22	0·06	–0·08, 0·21	0·06	–0·08, 0·21	0·08	–0·09, 0·25
Anaemia	–0·44	–0·55, –0·34	–0·44	–0·54, –0·35	–0·44	–0·54, –0·35	–0·44	–0·54, –0·35

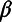
 , average annual percentage change.

**Fig. 1:**
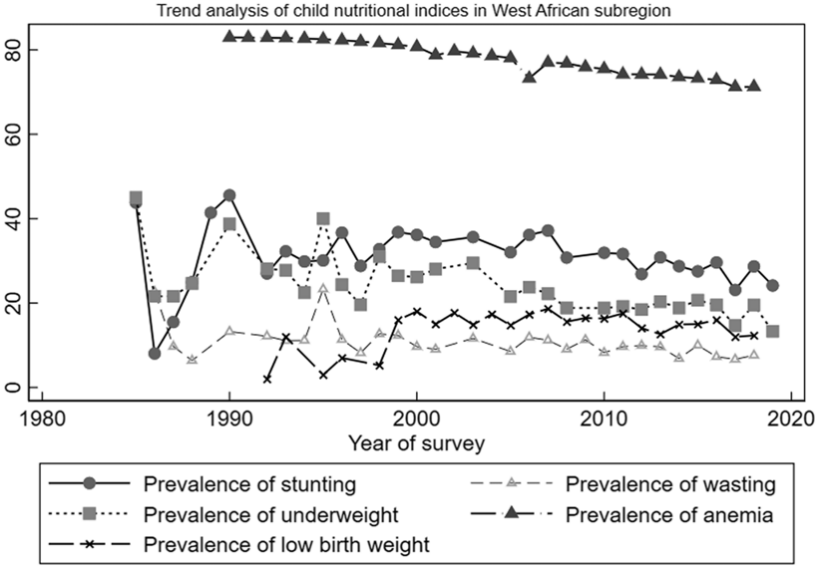
Trend analysis of low birth weight and child undernutrition in West Africa for the period 1985–2019

Figure 1 depicts the trend analysis of LBW and child undernutrition (stunting, wasting, underweight and anaemia) in WA. The trend analysis of the outcomes for each of the West African countries is depicted in Appendix A1–A5. Overall, the regional trends of undernutrition and LBW were not different from what was observed at the country levels. Our results identified three distinct periods (1986–1990, 1993–1996 and 1997–2000) of sharp increases in the prevalence of wasting, stunting and underweight. Anaemia prevalence, however, remained relatively stable during the period. With regard to LBW, a sharp decrease was observed between 1993 and 1996 followed by a sharp increase between 1997 and 2000. Stunting prevalence saw a rapid decline and increase between 1985 and 1990. After the year 2000, prevalence of all outcomes except LBW started to decline with some fluctuations but never close to prevalence levels observed in 2000. Prevalence of LBW, however, remained somehow stable after 2000 up till after 2010 when it started to decline.

**Fig. 2:**
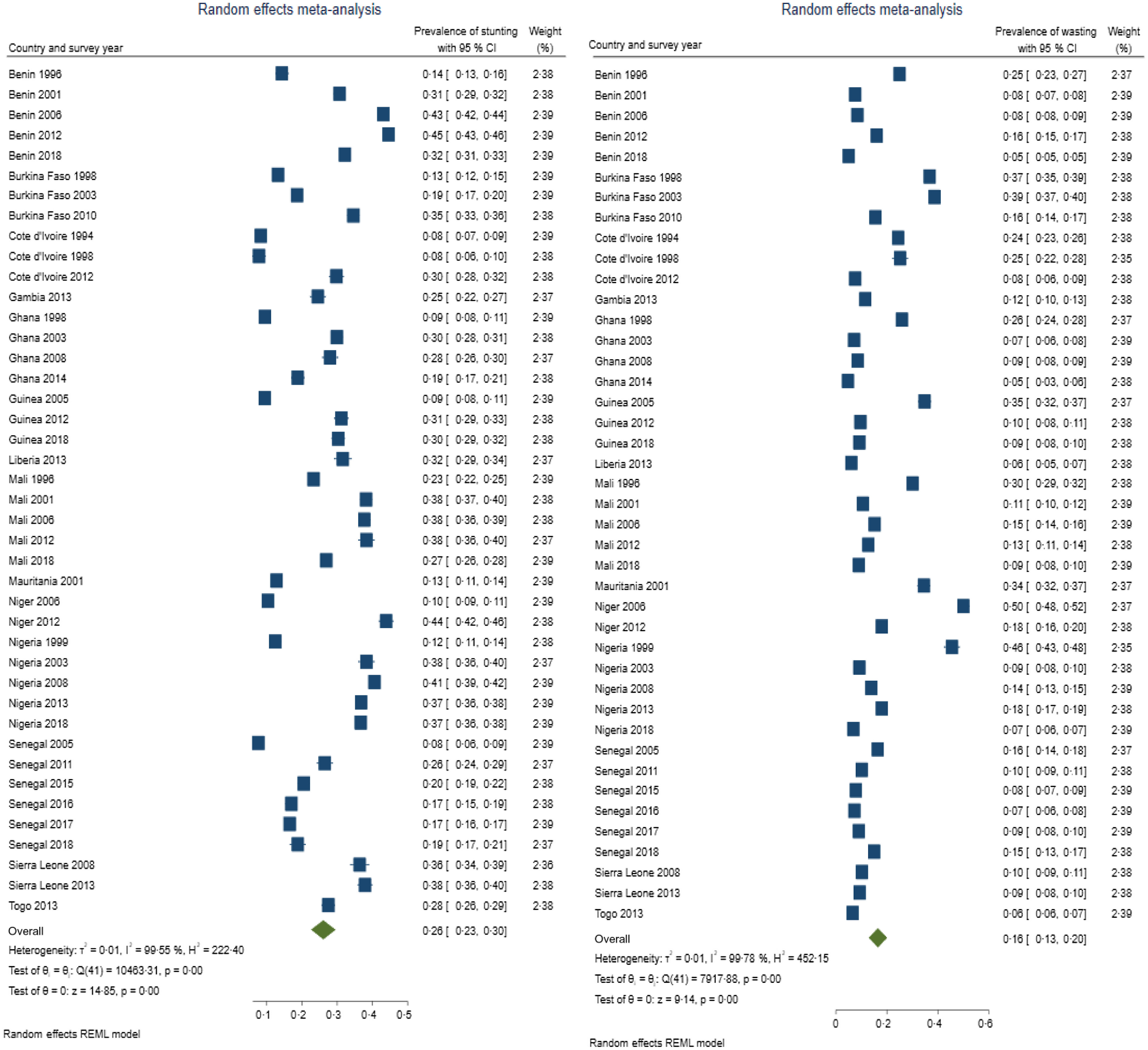
Forest plot showing summary prevalence estimate of stunting (A) and wasting (B) for West Africa

**Fig. 3:**
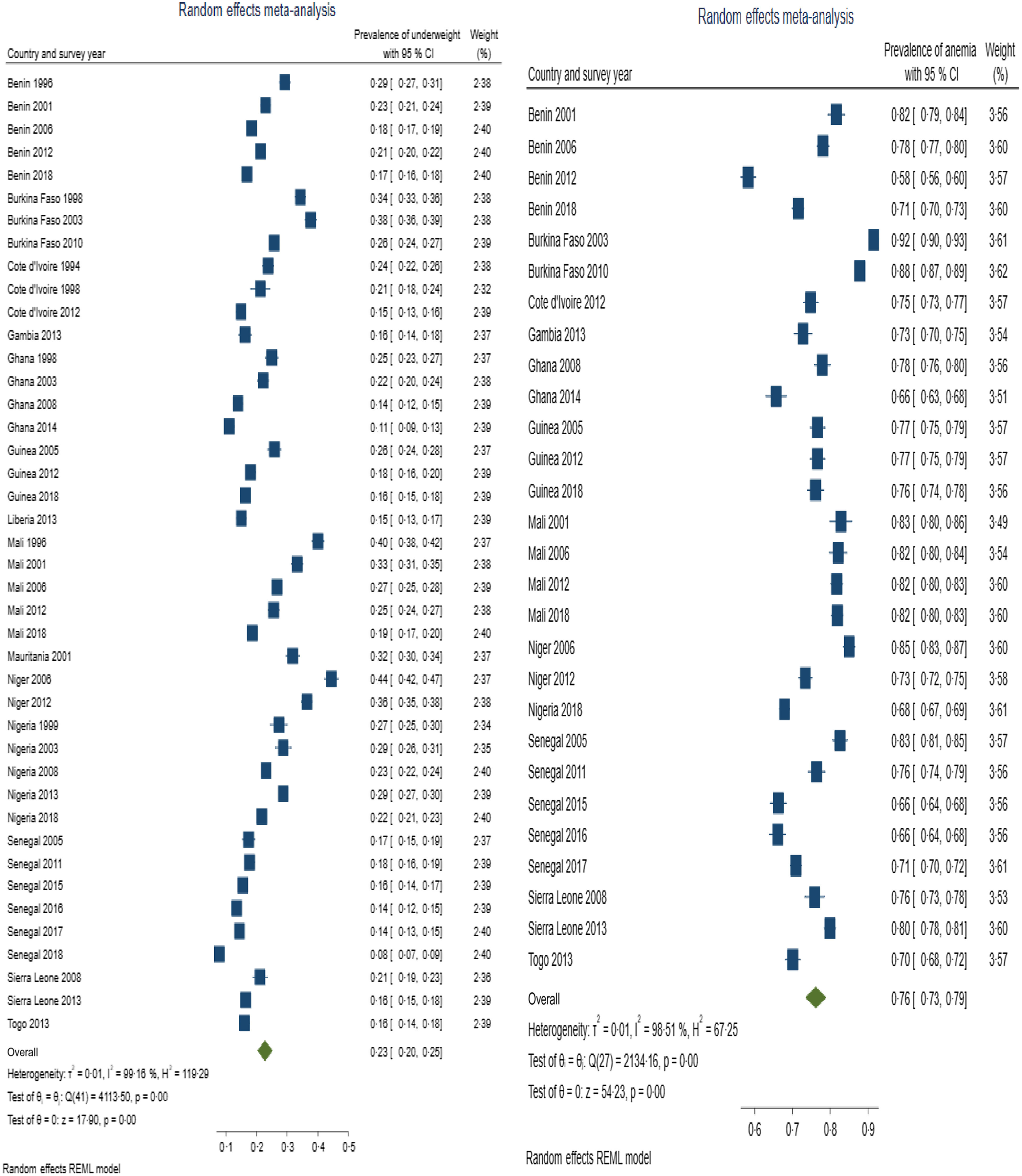
Forest plot showing summary prevalence estimate of underweight (A) and anaemia (B) for West Africa

**Fig. 4:**
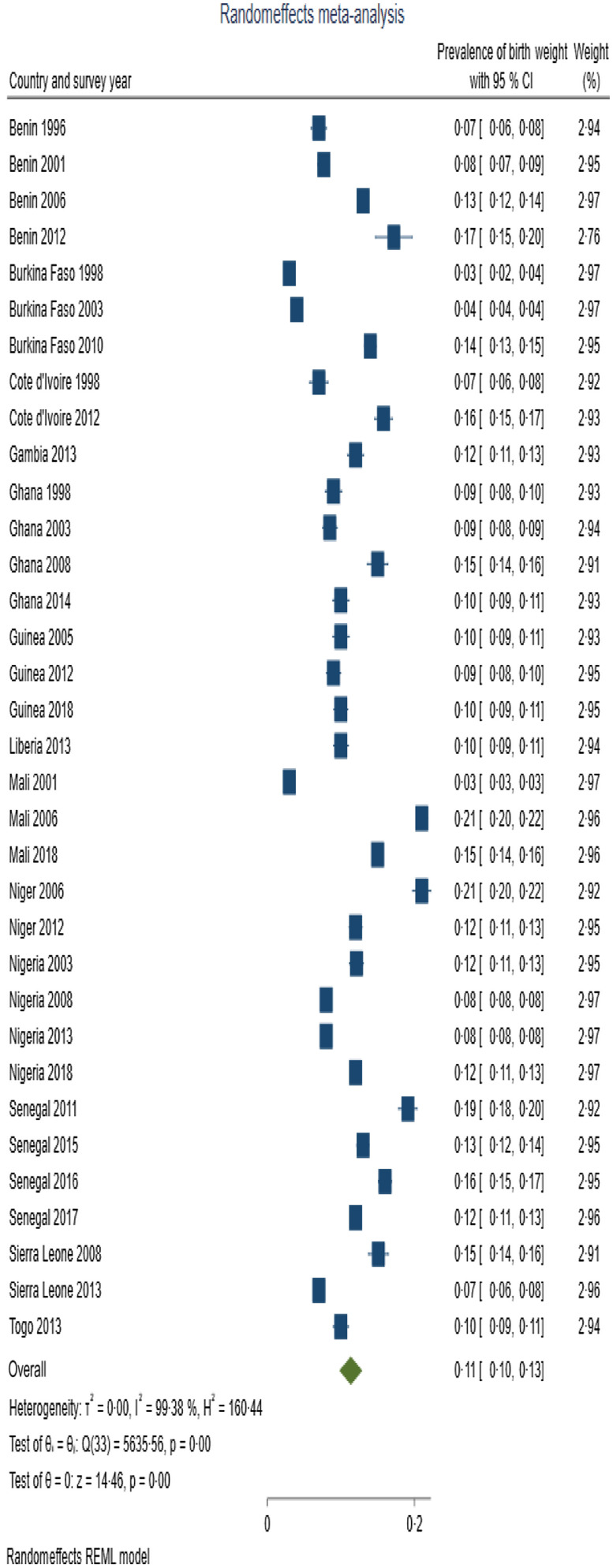
Forest plot showing summary prevalence estimate of low birth weight for West Africa

Table 3 presents summary prevalence estimates of the outcomes of interest for WA. The summary prevalence estimate of stunting, wasting, underweight, anaemia and LBW for WA was 26·1 % (95 % CI 22·5, 29·6), 16·4 % (95 % CI 12·8, 20·0), 22·7 % (95 % CI 20·2, 25·3), 76·2 % (95 % CI 73·3, 79·0) and 11·3 % (95 % CI 9·7, 12·9), respectively. The forest plots are depicted in Fig. 2–4. For all the outcomes, we observed very high degree of heterogeneity in the prevalence estimates combined (I^2^ ranged from 98·73 to 99·61 %). The Eggers test for small-study effect and publication bias was statistically significant for all the outcomes except stunting (z = 1·90, *P* = 0·0573) and anaemia (z = –1·36, *P* = 0·1734) indicating the absence of publication bias for these two outcomes. The summary prevalence estimates did not change in the sensitivity analysis using the empirical Bayes estimator. The country and year of survey contributed 75·1 %, 45·6 %, 18·5 %, 16·5 % and 4·9 % of the between-study heterogeneity in the prevalence estimate computed for underweight, stunting, wasting, anaemia and LBW, respectively. For LBW, the *P*-value was, however, greater than 0·05.

**Table 3:**
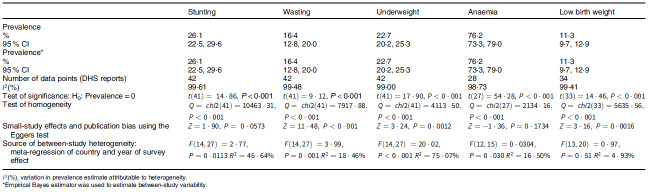
Summary prevalence estimate of low birth weight and childhood undernutrition in West Africa

Table 4 presents the country-specific and year-cohort summary prevalence estimates for the outcomes of interest. Over the study period (1985 to 2019), Sierra Leone recorded the highest prevalence of stunting (37·3 %, 95 % CI 35·8, 38·9) with Niger recording the highest prevalence of underweight (40·4 %, 95 % CI 32·5, 48·2), wasting (34·0 %, 95 % CI 2·6, 65·4) and LBW (16·5 %, 95 % CI 7·7, 25·3). Burkina Faso recorded the highest prevalence of anaemia (89·6 %, 95 % CI 86·0, 93·2). Cote d’Ivoire and Senegal, respectively, recorded the lowest prevalence of stunting (15·3 %, 95 % CI 2·9, 27·7) and underweight (14·3 %, 895 % CI 11·2, 17·5). Sierra Leone recorded the lowest prevalence of wasting (9·8 %, 95 % CI 8·9, 10·6) with Ghana recording the lowest prevalence of anaemia (71·8 %, 95 % CI 59·9, 83·8). Burkina Faso recorded the lowest prevalence of LBW in the subregion (7·0 %, 95 % CI 1·6, 12·4). Niger, however, recorded the smallest number of data points (*n* 4) in the analysis (Table 1). Underweight and wasting prevalence in the subregion declined from 28·7 % and 30·4 % during the period 1990–1999 to 18·4 % and 10·2 % during the period 2010–2019, respectively. Anaemia prevalence also declined from 81·4 % during the period 2000–2009 to 73·3 % during the period 2010–2019. LBW and stunting prevalence, however, increased from 6·5 % and 12·7 % during the period 1990–1999 to 12·3 % and 29·8 %, respectively, during the period 2010–2019.

**Table 4:** Country-specific and year-cohort summary prevalence estimate for low birth weight and childhood undernutrition for the period 1985–2019

Country/period	Stunting		Underweight		Wasting		Anaemia		Low birth weight	
	Prevalence (%)	95 % CI	Prevalence (%)	95 % CI	Prevalence (%)	95 % CI	Prevalence (%)	95 % CI	Prevalence (%)	95 % CI
Benin	33·3	23·4, 42·5	21·7	18·1, 25·3	12·3	7·7, 16·9	72·4	63·2, 81·6	11·1	7·3, 14·8
Burkina Faso	22·1	10·1, 34·2	32·5	25·1, 40·0	30·3	14·7, 46·0	89·6	86·0, 93·2	7·0	1·6, 12·4
Cote d’Ivoire	15·3	2·9, 27·7	19·9	13·7, 26·1	19·0	6·1, 31·9	*		11·4	2·8, 20·0
Ghana	21·5	11·1, 31·9	18·0	11·6, 24·3	11·5	5·0, 18·0	71·8	59·9, 83·8	10·6	8·0, 13·1
Guinea	23·6	8·5, 38·8	20·0	15·0, 25·0	17·8	6·1, 29·6	76·5	75·3, 77·6	9·6	9·0, 10·3
Mali	32·9	26·3, 39·4	28·8	21·7, 35·8	15·5	9·5, 21·5	82·0	81·0, 82·9	13·0	1·3, 24·7
Niger	27·1	5·8, 60·0	40·4	32·5, 48·2	34·0	2·6, 65·4	79·2	67·8, 90·6	16·5	7·7, 25·3
Nigeria	33·0	23·8, 42·1	25·8	22·8, 28·8	18·5	11·8, 25·2	*		10·0	8·0, 12·0
Senegal	17·8	12·9, 22·6	14·3	11·2, 17·5	10·8	8·5, 13·1	72·5	66·6, 78·3	15·0	12·2, 17·8
Sierra Leone	37·3	35·8, 38·9	18·6	14·0, 23·3	9·8	8·9, 10·6	78·0	74·1, 81·9	11·0	3·1, 19·0
1990–1999	12·7	8·7, 16·7	28·7	23·9, 33·5	30·4	25·5, 35·3	*		6·5	3·4, 9·5
2000–2009	27·2	19·9, 34·6	26·2	22·2, 30·2	18·9	14·1, 23·6	81·4	78·0, 84·9	11·5	8·3, 14·8
2010–2019	29·8	25·9, 33·6	18·4	15·9, 21·0	10·2	8·6, 11·8	73·3	69·5, 77·0	12·3	10·8, 13·8

* Prevalence estimate or corresponding standard error missing. The meta-analysis was conducted for countries with at least two DHS reports, and hence estimates are not provided for Gambia and Mauritania. Estimates are not provided for Togo and Liberia because either prevalence estimates are not available for an outcome measure or standard error for prevalence estimate is missing. Estimates are not provided for the period 1985–1989 due to prevalence estimates missing corresponding standard error or CI for almost all countries. The country estimates were adjusted for year of survey.

## Discussion

We observed a marginal decline in the prevalence of stunting, wasting, underweight and anaemia among children under 5 years of age and an increase in LBW. The summary prevalence estimates of wasting (16·4 %) and underweight (22·7 %) were deemed to be very high based on the cut-off values for public health significance proposed by de Onis *et al.* ^(19)^ In five countries (Benin, Burkina Faso, Mali, Niger and Nigeria), the underweight prevalence exceeded the regional average of 20·1 %^(2)^. In Niger (34·0 %) and Burkina Faso (30·3 %), the wasting prevalence exceeded the global average (6·9 %)^(1)^ by more than fourfolds. The overall prevalence of wasting and stunting in WA exceeded the global average of 6·9 % and 21·3 %, respectively^(1)^.

The child undernutrition trends observed during the three distinct periods (1986–1990, 1993–1996 and 1997–2000) could be explained by a complex set of macro-level factors including political, economic, social and environmental factors. Famine as a result of the drought observed in WA in the 1980s^(20)^ could explain the findings during the period 1986–1990. High levels of child undernutrition have been reported in drought-affected population in LMIC^(21,22)^. Implementation of structural adjustment programmes in the 1980s in developing countries including West African countries might have also contributed to the increase in undernutrition in the region^(23)^. SSA accounted for the largest percentage of structural adjustment programmes^(24)^ with the programme documented to have adversely affected healthcare^(25)^ and associated with increased infant mortality in SSA^(26)^. A systematic review by Thomson *et al.* ^(27)^ also reported structural adjustment programmes to have resulted in poor child health indicators in developing countries.

Our observations between the period 1993 to 1996 could be explained by the worsening macroeconomic conditions in many West African countries during that period, especially the 1994 devaluation of the CFA franc which could have contributed to the spikes in undernutrition levels observed in the mid-1990s. Currency devaluation has been associated with high food prices, poor diet quality, reduced food intake and poor healthcare with consequences for birth weight, and child nutrition and growth^(28,29)^. Our results also showed a sharp increase in the prevalence of stunting, wasting and underweight as well as LBW just before the turn of the millennium. The world economic crises in the late 1990s might have accounted for the observed trend. A study conducted in East Asia reported an increase in the prevalence of LBW and child underweight during the world economic crises^(30)^.

After the millennium, prevalence of child undernutrition in WA witnessed a decline with very marginal increases. A number of factors might have contributed to the improving trends of undernutrition after the millennium. Food availability is an important determinant of child nutrition. The Economic Community of West African States (ECOWAS) Regional Agriculture Policy (ECOWAP) and the Regional Agricultural Investment Plan (RAIP) were implemented in the 2000s. These policies led to significant improvement in agricultural productivity, especially of staples such as cereals, roots and tubers that are consumed in all West African countries^(31,32)^. These policies might have contributed to the reduction in prevalence of undernutrition observed after the millennium. By 2010, food deficit in WA had reduced to 79 kcal/capita/d from 106 kcal/capita/d in 2000^(31)^. Another possible reason for the improvements is the increase in gross domestic product (GDP) growth rates observed in WA in the 2000s^(33)^. Economic growth is documented to decrease child undernutrition. Smith and Haddad^(34)^ showed that undernutrition can be reduced by up to 50 % with increase in per capita income. Other studies have also found increase in gross domestic product to decrease prevalence of stunting and underweight^(35–37)^.

The observed average annual percentage decline in wasting, underweight and anaemia could be attributed to steady improvements in agricultural productivity in the subregion from the 1980s up to present time. A Food Crisis Prevention Network (RPCA) report in 2016 indicated that although the population of WA has more than doubled in the past three decades to more than 370 million people in 2015, there has been steady improvements in agricultural productivity since the 1980s at an impressive rate of 2·6 % per year^(38)^. According to the report, the improvement in agricultural productivity has been able to support the increasing population demands and led to significant improvements in dietary practices in the region^(38)^.

Stunting was the most prevalent form of child undernutrition in WA and was consistent with previous studies^(1,39)^. Poverty, conflict and socio-economic deprivation strongly drives childhood stunting in many developing countries and could explain the findings of our study. Droughts, floods, crop-destroying pests, economic crises and conflicts are recurring problems in WA and impacts food and nutrition systems^(38,40)^. Also, countries in the Sahel region (Mauritania, Mali, Burkina Faso, Niger and Nigeria) are very fragile due to terrorist activity^(39)^ which deepens poverty and food insecurity. Drought is also a major problem in the Sahel countries owing to climate change and further exacerbates the stunting problem.

The steady increase in prevalence of LBW after 2000 and the observed increase in average annual percentage change in LBW prevalence in WA can be attributed to low women empowerment in West African countries which affects maternal nutrition significantly. Low levels of women empowerment have been associated with maternal undernutrition and LBW in LMIC^(41,42)^. In WA, women’s economic empowerment has been stalled by low educational attainment among women, cultural practices that place the burden of domestic work on women and girls, and early marriage and teenage pregnancy^(41,43)^. Half of SSA women have been found to have no basic literacy skills^(44)^. Also, about 74 % of working women in SSA are in low productivity agriculture and informal employment, compared with 61 % of men^(45)^. There has been several policies and programmes to empower women in WA^(46,47)^ and have great potential for improving dietary diversity of WA households^(41)^. In spite of these interventions, low women empowerment still persists in WA with challenges for the health and well-being of women including access to nutritious foods. According to a study conducted in Burkina Faso^(48)^,although mothers were aware they were the focus of nutrition interventions, they were not empowered to make the necessary nutrition decisions owing to such decisions having the tendency to cause marital problems. The situation according to the authors contributes to poor maternal nutrition within the population. In addition to low women empowerment, widespread social deprivation in most West African countries also explains the observed increase in average annual percentage change in LBW prevalence. Several studies have reported strong association between birth weight and socio-economic status^(49–51)^.

Teenage pregnancy has been on the rise in several West African countries in recent years owing to poor socio-economic condition and can also explain the LBW trend observed in our study. Low maternal age is a well-documented risk factor for LBW^(52–54)^. In most West African countries, the problem of early marriage still persists, especially in areas dominated by Muslims which further compound the teenage pregnancy problem. It is therefore not surprising that Niger, a country with the highest fertility rate in Africa^(55)^ and also having the lowest age for marriage and childbearing^(56)^, had the highest prevalence of LBW in the present study.

We found prevalence of anaemia in WA to be about twice the global prevalence estimate of 41·5 %^(4)^ and is more prevalent in Burkina Faso (89·6 %). This finding can be explained by the rising hidden hunger in West African countries. Deficiency of essential vitamins and minerals, termed hidden hunger, deprive children of vitality throughout the life course, compromises their health and well-being, and inhibits their growth and development^(40)^. It has been reported that three in four children in West and Central Africa between the ages of 6 and 23 months are not eating foods from the minimum number of food groups to support their rapid growth^(40)^. The high prevalence of childhood anaemia in WA could also be attributed to a number of factors including malaria and soil-transmitted helminth infections, and hereditary diseases such as sickle cell anaemia which are all very prevalent in SSA^(9,57)^.

### Validity issues

The use of prevalence estimates from DHS for the study which are nationally representative and have high response rates inspires confidence in our summary estimates. This is because random and systematic errors that are the shortcomings of individual studies in meta-analytic studies are minimised greatly. DHS surveys also uses standardised data collection procedures across countries and allows comparability across populations cross-sectionally and over time^(58)^. In addition, DHS facilitates epidemiological research that are focused on monitoring of prevalence, trends and inequalities^(58)^.

The study provides summary prevalence estimate for stunting, wasting, underweight, anaemia and LBW for WA using the DerSimonian–Laird method which accounts for between- and within-country variability. We adjusted for the standard error of the summary prevalence estimate using the Knapp–Hartung adjustment which improves precision of the estimated between-study variance associated with using small samples. A limitation of the DerSimonian–Laird method is that it could lead to an underestimation of the true between-study variance, if the variability is large and the number of studies are small. Mindful of the limitation of the method, we conducted a sensitivity analysis using empirical Bayes estimator^(59)^ which tends to be less biased than other random effects methods, but also less efficient than the DerSimonian–Laird method^(60)^ to assess robustness of our findings. Also, in the trend analysis, we used different model specification (random effects, mixed effects and generalised estimating equation) to estimate the average annual percentage change in prevalence of the outcomes studied to improve reliability of the effect sizes estimated. The prevalence estimates computed for the respective countries over the study period were adjusted for year of survey to remove the influence of the lack of consistency in the survey years in the countries and render the estimates comparable across the countries.

This study, however, has some limitation. First, most of the prevalence estimates obtained from the World Bank data repository did not include the total sample size used to generate the estimates, as well as the accompanying standard errors and CI. Also, in a few cases, the prevalence estimates extracted from the DHS reports did not have the corresponding standard errors. In addition, for some survey years in some countries, estimates were missing for some of the outcomes. As a result, we could not use these estimates in the meta-analysis, which could bias the summary prevalence estimates computed. We did, however, investigated publication bias in the meta-analysis to account for the missing data.

## Conclusions

In conclusion, we explored the trend and quantified the prevalence of stunting, wasting, undernutrition, anaemia and LBW and provide the evidence base for assessing the effectiveness of national and regional interventions that have been implemented over the last four decades for tackling child undernutrition and LBW in WA. Even though we observed a decline over the study period in child undernutrition, the trends in recent times may not be sufficient to help reach the Global Nutrition Targets 1, 4 and 6 in 2025 and requires new and integrated set of nutrition-specific and sensitive interventions. To reach these global nutrition goals, governments should put in place policies and programmes aimed at socio-economic development to help reduce poverty and socio-economic deprivation as well as empower women.

## Supplementary material

For supplementary material accompanying this paper visit https://doi.org/10.1017/S1368980022000155. click here to view supplementary material
