# Supplementary figures and images for: Secular trends in low birth weight and child undernutrition in West Africa: evidence from complex nationwide surveys, 1985–2019
Source: Public Health Nutr. 2022 Jan 18;25(9):2358–70. doi: 10.1017/S1368980022000155 (PMC9991671; doi:10.1017/S1368980022000155)

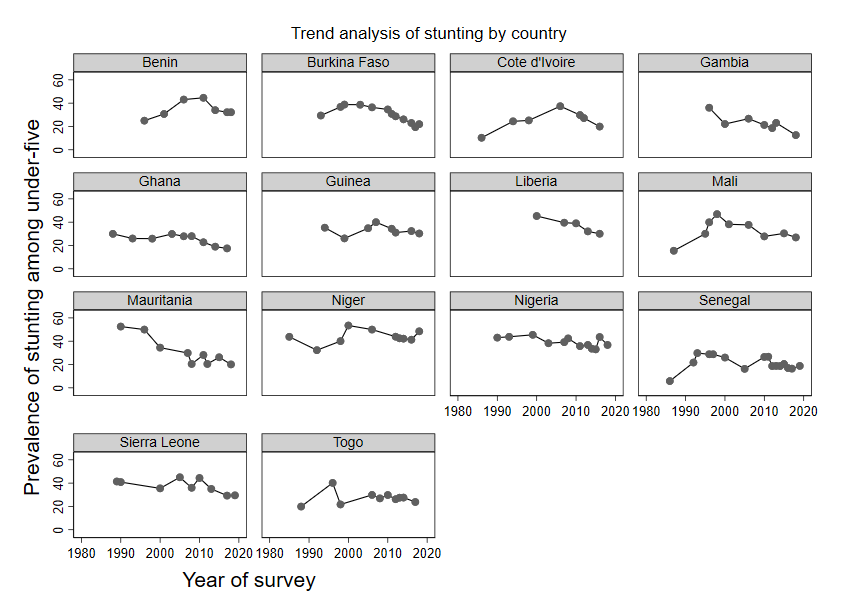


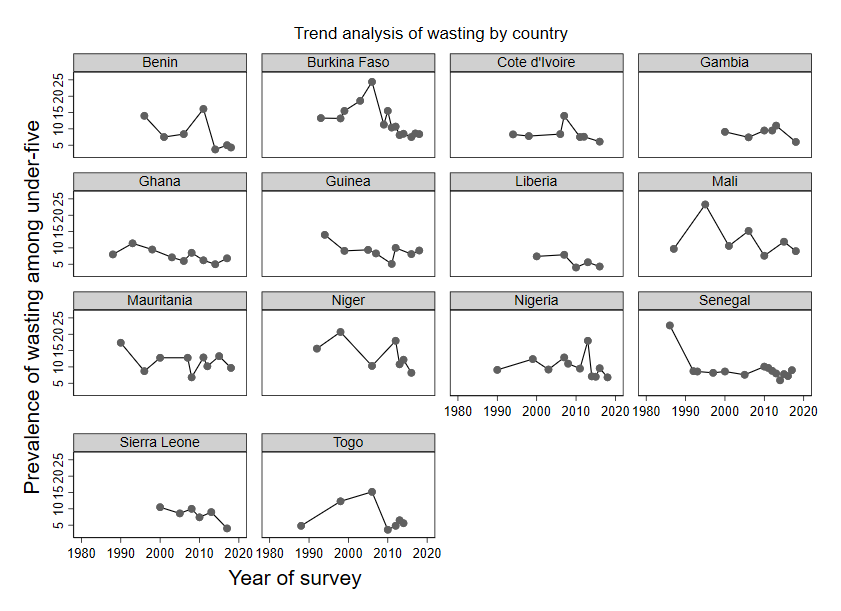


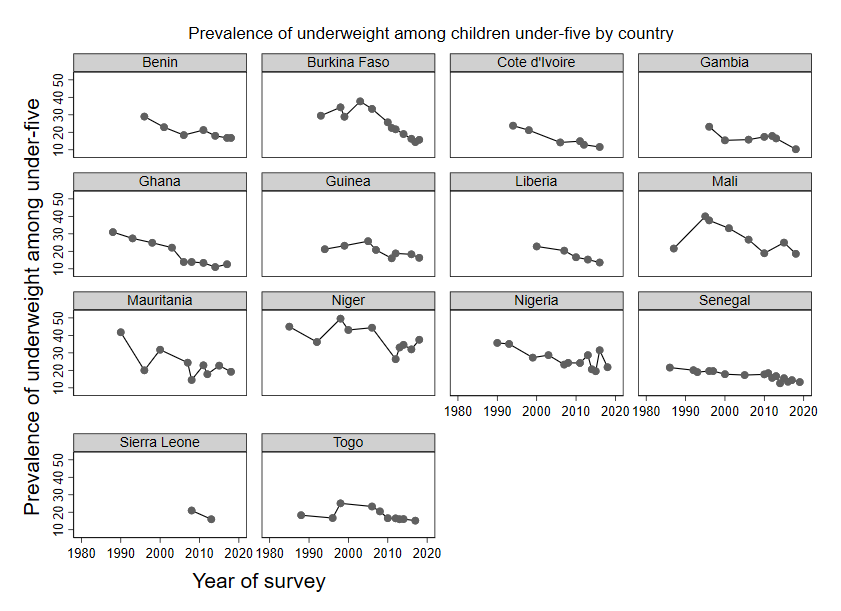


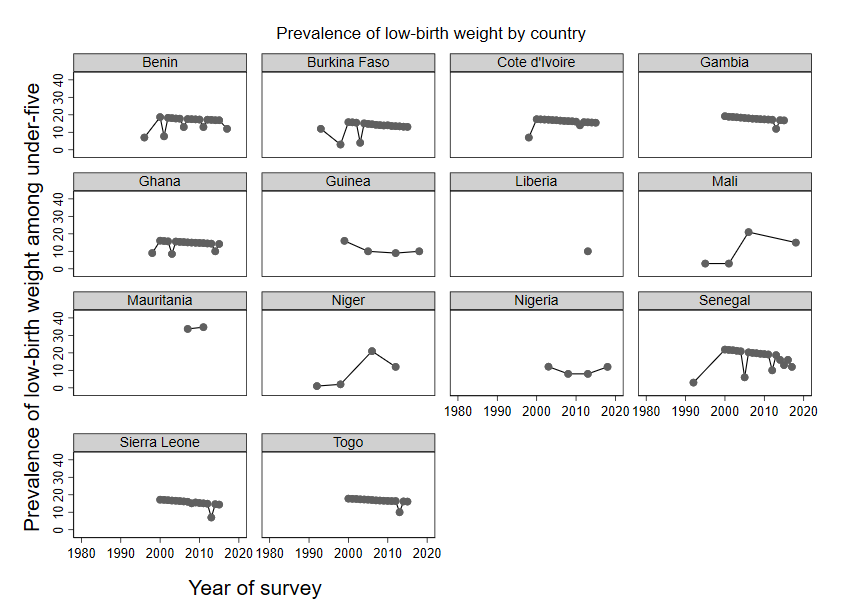


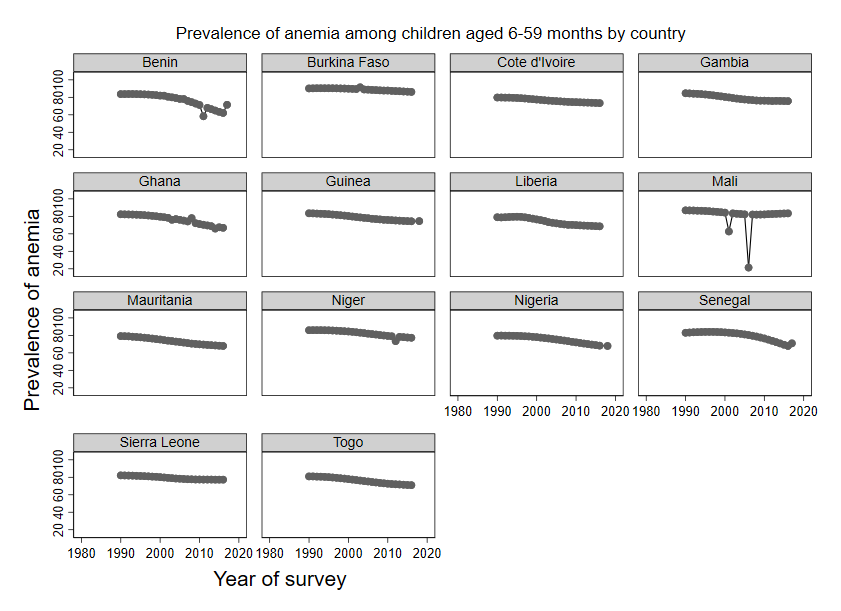

Supplement: Supplementary file 1 [file S1368980022000155sup001.docx]
